# Swelling-Activated, Soft Mechanochemistry in Polymer Materials

**DOI:** 10.1021/acs.langmuir.2c02801

**Published:** 2023-02-27

**Authors:** Friederike Katharina Metze, Sabrina Sant, Zhao Meng, Harm-Anton Klok, Kuljeet Kaur

**Affiliations:** Institut des Matériaux and Institut des Sciences et Ingénierie Chimiques, Laboratoire des Polymères, École Polytechnique Fédérale de Lausanne (EPFL), Station 12, CH-1015 Lausanne, Switzerland

## Abstract

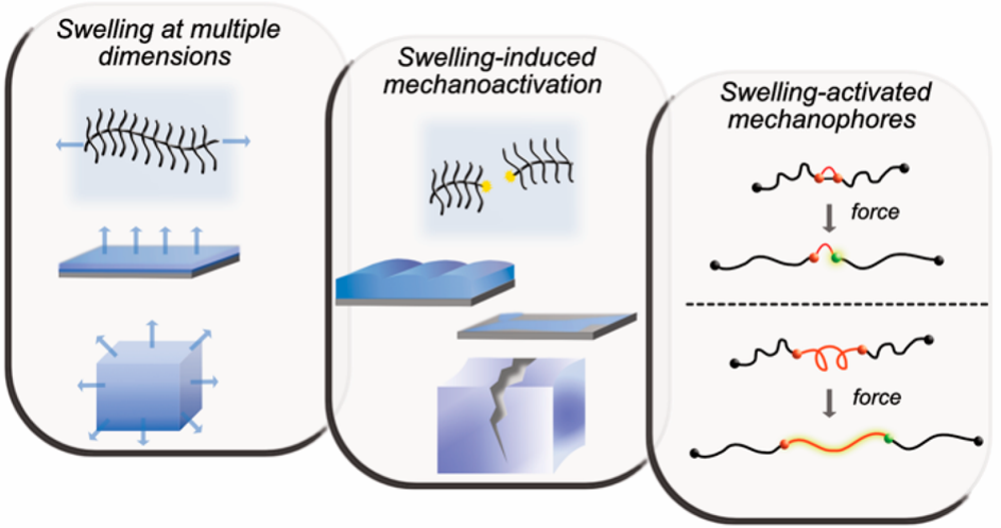

Swelling in polymer materials is a ubiquitous phenomenon. At a molecular level, swelling is dictated by solvent–polymer interactions, and has been thoroughly studied both theoretically and experimentally. Favorable solvent–polymer interactions result in the solvation of polymer chains. For polymers in confined geometries, such as those that are tethered to surfaces, or for polymer networks, solvation can lead to swelling-induced tensions. These tensions act on polymer chains and can lead to stretching, bending, or deformation of the material both at the micro- and macroscopic scale. This Invited Feature Article sheds light on such swelling-induced mechanochemical phenomena in polymer materials across dimensions, and discusses approaches to visualize and characterize these effects.

## Introduction

The effects of mechanical force on macromolecules have been known since the early 1930s starting with a report by Staudinger and Heuer, who observed a reduction in molecular weight of polystyrene upon mastication.^1^ This was interpreted as ruptures along the polymer backbone into smaller fragments under the influence of an external mechanical force. Later, Kauzmann and Eyring refined this idea by mathematically showing that force can alter reaction pathways by lowering the bond-dissociation energy of stretched bonds.^2^ These seminal works of the early 20th century paved the way toward a better understanding of the structure and properties of macromolecules. It is now well established that external force can cause homolytic carbon–carbon bond scission in the polymer backbone, which, if present in sufficient numbers, can lead to the deterioration of the mechanical properties of polymers.^3,4^ Over the past decades, the focus of the field of mechanochemistry has shifted from studying and understanding polymer degradation under mechanical force toward the design of polymer materials that are able to undergo mechanochemical transformations in a predictable and controllable manner. This has paved the way, e.g., to force reporting or self-healing materials. Key toward harnessing mechanical forces in a productive manner has been the development of mechanophores, which are force-sensitive molecular units that incorporate strategically weakened, mechanically labile bonds.^5−8^

The forces typically considered in polymer mechanochemistry cover a broad range from ∼100 pN to 10^5^ N.^5,9^ Nature, in contrast, makes use of forces that can be even weaker (10–100 pN) to control, for example, cellular adhesion, division, differentiation, and motility,^10,11^ as well as protein folding, enzymatic activity,^12^ and several other biological processes.^13^ While a lot of work has been done to study and understand the effects of strong forces on synthetic polymer materials, comparably little is known about the response of synthetic polymer materials to these weak forces. The term “soft mechanochemistry” has been coined to describe the effects of these weaker forces.^14^

Swelling by solvent uptake represents one strategy to apply a weak mechanical load to polymer materials. Swelling of polymer materials is described as the process of penetration of solvent molecules into a polymer matrix causing a change in the volume. In accordance with the Flory–Rehner theory, swelling can be seen as an equilibrium between the entropy of polymer chains and the enthalpy of mixing.^15,16^ When swollen in a good solvent, the polymer chains stretch due to favorable solvent–polymer interactions. In order to balance the decrease in entropy associated with the resistance of polymer chains to swelling-induced stretching, an elastic retractive force develops in the polymer network.

Solvent uptake and swelling can lead to mechanical instabilities in both natural and synthetic materials. In nature, many examples can be found, such as the opening and closing of pine cones caused by swelling-controlled changes in the morphology of their bilayered scales,^17^ or swelling-induced stretching, bending, and curling of articular cartilage.^18^ In synthetic materials, solvent uptake and swelling can cause buckling, wrinkling or delamination, and bending of thin polymer films.^19^ Solvent-driven expansion and contraction of soft and flexible polymer materials form the basis for artificial muscles, wearable electronics, and soft robots.^20,21^

In addition to driving changes in shape or volume, there is also increasing evidence that swelling of cross-linked polymer networks can lead to tension forces that are sufficiently strong to facilitate bond cleavage reactions.^22,23^ While a considerable and growing body of experimental evidence points toward swelling-driven or accelerated bond cleavage processes in polymer networks, there are many open questions as to the mechanistic origins of these processes. The aim of this paper is to provide perspectives toward a better understanding of the swelling-driven mechanochemical activation of polymers, as well as prospects to leverage these phenomena for the design of new responsive polymer materials. This paper is divided into two parts. First, a comprehensive overview will be provided of examples of polymer materials for which bond scission events have been observed upon solvent swelling. The second part of this paper will present several techniques, some of which have been used, and others that may be powerful complementary tools to observe and quantitatively study swelling-driven mechanochemical activation of cross-linked polymer networks. Finally, we address the challenges concerning the translation of forces at the molecular level and their characterization in the context of developing novel mechanoresponsive materials.

## Swelling-Induced Mechanochemical Activation in Multidimensional Polymer Materials

**Figure 1:**
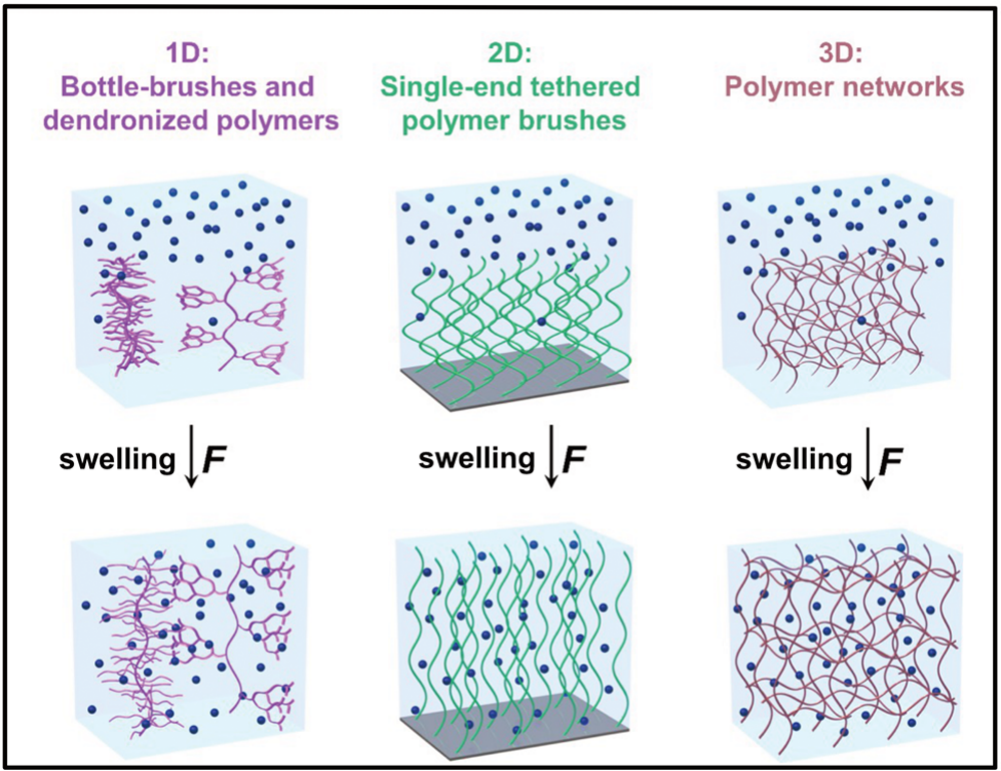
Swelling of various polymer materials induces a force that acts on polymer chains. The presented polymer materials can be divided into 1D, bottle-brushes and dendronized polymers; 2D, single-end tethered polymer brushes; and 3D, polymer networks.

Solvent-induced effects are observed in a variety of polymer materials. Here, these are categorized into three main types (Figure 1): one-dimensional systems comprising polymer chains in melt or solution, two-dimensional polymer thin films including both constrained (i.e., surface-attached) and free-standing films, and three-dimensional chemically cross-linked polymer networks.

### One-Dimensional Polymer Materials

#### Bottlebrush Polymers

**Figure 2:**
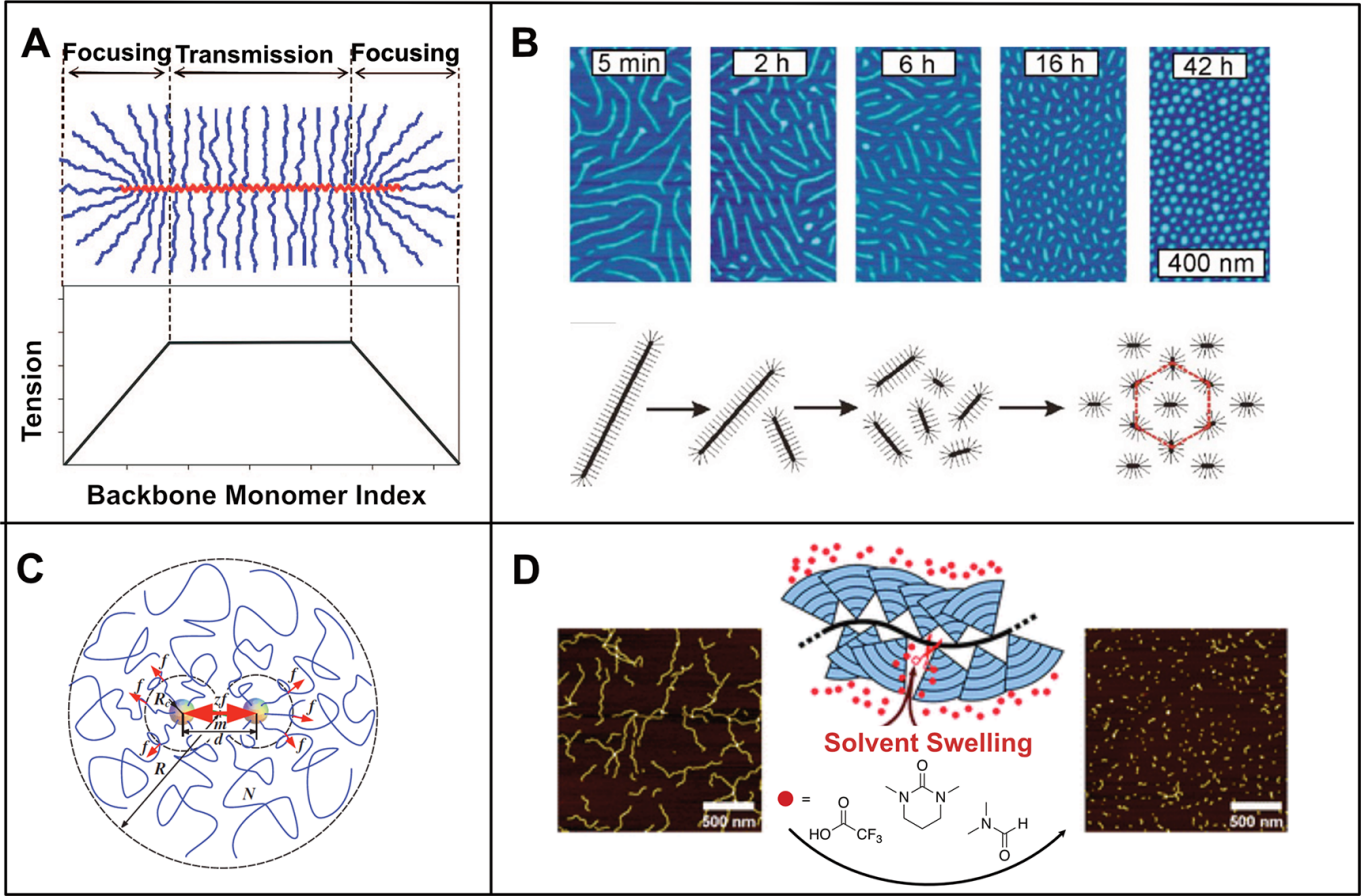
(A) Mechanical tension in the bottlebrush backbone originates in the focusing regions and is then transmitted along the backbone to the middle of the chain. (Reprinted with permission from ref (28). Copyright 2009 American Chemical Society.) (B) AFM micrographs of bottlebrush polymers on a mica surface in a water–propanol mixture undergoing backbone degradation over time. (Reprinted with permission from ref (27). Copyright 2006 Springer Nature.) (C) Representation of the force concentration in the spacer of dendronized polymers. (Reprinted with permission from ref (29). Copyright 2009 American Physical Society.) (D) Swelling-induced main chain scission of dendronized polymers of 5th generation. (Reprinted from ref (32) under the terms of the CC BY-NC 3.0 license.)

Bottlebrush polymers are branched polymers with long side chains grafted at regular intervals along the polymer backbone. They are characterized by the degree of polymerization of the side chains as well as the spacer separating neighboring side chains (Figure 2A). The physical properties and mechanical behavior of these systems are of great interest due to their structural similarity to biomacromolecules like aggrecans and mucin.^24−26^ In 2006, Matyjaszewski and co-workers reported the fragmentation of a densely grafted bottlebrush polymer that was adsorbed on a substrate exposed to a water/isopropanol mixture. This was a result of the backbone extension as a consequence of attractive interactions between the side-chains and the substrate (Figure 2B).^27^ High substrate surface energy induces the spreading of side-chains by maximizing their contact with the substrate resulting in the extension of the backbone beyond its physical limits leading to rupture of carbon–carbon bonds. Subsequent theoretical studies showed that the solvent-induced tension in the backbone of bottlebrush polymers is estimated to increase from ∼4 pN in the melt (nonsolvent state) to ∼50 pN in an athermal solvent.^28^ A significant amplification of backbone tension of up to ∼2 nN was estimated to be achievable upon their adsorption onto a substrate, which results in spontaneous multiple carbon–carbon bond cleavages. Overall, these studies highlight the role of solvent–polymer interactions in the origin of weak tensions in the bottlebrush polymers ultimately leading to “fatal fractures” at solvent–solid interfaces.

#### Dendronized Polymers

The backbone tension in branched macromolecules can be further amplified by increasing the steric bulk of the side chains.^28^ In a theoretical study, Panyukov, Sheiko, and Rubinstein showed that *star* or *pom-pom* (z-arm) shaped side-chains can generate high backbone tensions on the order of nanonewtons in solution (Figure 2C).^29^ The weak tension (on the order of pN) caused by osmotic repulsions of *z* individual side arm chains is focused at a single backbone spacer resulting in a *z*-fold increase in spacer tension (on the order of nN) in solution. They further concluded that the tension in a spacer is dependent on the solvent quality and ranges from ∼4 pN in melt to ∼100 pN for theta solvents and can even reach up to 2 nN for short spacers.

Dendronized polymers are *pom-pom*-like architectures where side chains consist of highly branched structures or dendrons attached to a linear backbone. These are synthesized using either macromonomer or “graft-onto” routes including both convergent and divergent approaches.^30^ Zhang and co-workers reported main chain scission in a generation 5 (*g* = 5) dendronized polymer, which was synthesized by a divergent approach that involved a series of protection–deprotection steps with charged intermediates.^31^ The observed backbone scission was believed to be the result of increased steric crowding of the neighboring dendrons, which was further exaggerated by Columbic repulsions of peripheral charges on the dendron branches. However, when Schlüter and co-researchers used an alternative synthetic pathway, involving charge-neutral intermediates, dendronized polymers with *g* = 6, 7, and 8 were synthesized contradicting the original hypothesis pertaining to Coulombic repulsion-induced chain scissions.^32^ Furthermore, it was observed that chain scission was exclusive to polaraprotic solvents chemically similar to dendrons and was most facile for dendronized polymers of *g* = 5 (Figure 2D). The authors concluded that the main chain scission in dendronized polymers is a result of swelling-induced mechanical stress specific to *g* = 5. The rationale behind this observation was that the separation between neighboring dendrons in the fifth generation allows facile solvent penetration resulting in chain reorganization and subsequent amplification in the backbone tension. Swelling of less crowded dendronized polymers (*g* < 5) induces a small strain in the backbone which can be offset by accommodation of solvent molecules and swelling-induced chain rearrangements within the dendritic branch work. Dendronized polymers with *g* > 5 swell to only a small degree as the side chain crowding causes an impenetrable steric barrier for the solvent molecules, which therefore show no scission.

### Two-Dimensional Polymer Materials

Thin polymer films adhered to a substrate are often prepared by plasma-assisted vapor deposition methods, spin or dip casting (for surface-attached thin films), or surface-initiated (controlled) radical polymerization techniques (for polymer brushes). These films find various applications as smart surfaces for sensing and actuation, protective coatings, drug delivery, and more.^33,34^ The applications of these polymer films are dependent on their structure and properties, which are a direct consequence of their swollen state. Compared to the free-standing polymer gels and thin films, surface-attached thin films show confined swelling in the direction normal to the surface,^35^ making them interesting candidates for studying swelling-induced effects.

#### Polymer Brushes

The term polymer brush refers to an arrangement of densely grafted polymer chains on a substrate extending in a direction perpendicular to the surface. Polymer brushes can be prepared via the grafting-to method (where pre-synthesized polymer chains are attached to the substrate), or via the grafting-from strategy (where polymer chains are grown *in situ* from an initiator-modified substrate). Polymer brushes prepared via the grafting-from method are mostly synthesized using radical polymerization techniques, which includes free radical polymerization, as well as various controlled radical polymerization chemistries, such as atom transfer radical polymerization (ATRP), reversible addition–fragmentation chain-transfer (RAFT) polymerization, and nitroxide-mediated polymerization (NMP).^36,37^ The conformation of chain-end anchored polymer chains can vary from a “mushroom” to “brush-type” structure, depending on the grafting density, polymer molecular weight, solvent quality, and possible polymer–substrate interactions.^38,39^

**Figure 3:**
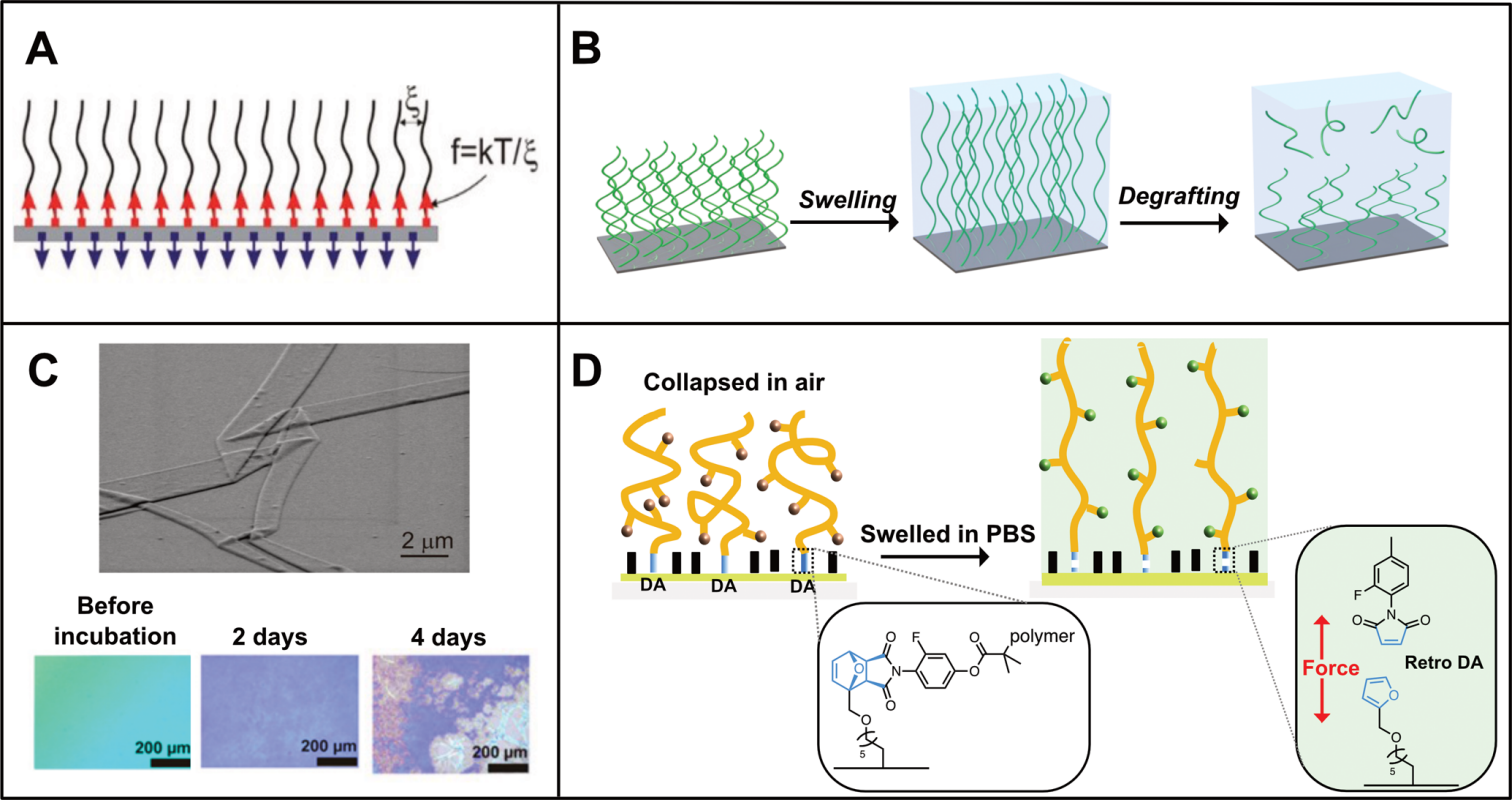
(A) Tension at the brush–substrate interface generated through tethering and steric repulsion. (Reprinted with permission from ref (40). Copyright 2011 American Chemical Society.) (B) Schematic illustration of the swelling and subsequent degrafting process of polymer brushes, which results in a gradual decrease in grafting density. (C) (top) Scanning electron microscopy (SEM) image of densely grafted PPEGMA brushes after 7 days of incubation in cell culture medium at 37 °C. (Reprinted with permission from ref (41). Copyright 2008 American Chemical Society.) (bottom) Optical micrographs of PPEGMA brushes incubated in cell culture medium at different time points. (Reprinted with permission from ref (43). Copyright 2016 American Chemical Society.) (D) Retro-Diels–Alder induced by solvent swelling. (Reproduced with permission from ref (46). Copyright 2015 American Chemical Society.)

In a single polymer chain tethered to a solid substrate, the number of allowed configurations is decreased and tension is generated in the bond(s) tethering the chain to the substrate. The steric repulsion of neighboring chains forces polymer brushes into an extended chain conformation causing an additional (but smaller) tension, which depends on the interchain distance or grafting density (Figure 3A). The resulting tension in polymer brushes is in the order of several piconewtons (pN) and not significant to cleave covalent bonds.^40^

Swelling, however, allows the polymer chains to extend even further from the surface, amplifying the tension at the polymer substrate interface. These swelling-induced forces in turn can impact the reactivity of the bonds (ester, amide, and siloxane bonds) tethering the polymer brushes to the surface. It is believed that these mechanical stresses can lower the energy barrier required for a chemical reaction such as, for example, hydrolysis to proceed. The cleavage of polymer chains from an underlying surface is known as degrafting, and has been demonstrated for a range of polymer brushes that have been grafted from surfaces using initiators with hydrolytically labile bonds upon exposure to aqueous media (Figure 3B). One of the first reports that was published in 2008 showed that poly(poly(ethylene glycol) methacrylate) (PPEGMA) brushes detached from silica surfaces upon incubation in cell culture medium.^41^ The formation of wrinkled structures was observed by scanning electron microscopy (SEM) analysis (Figure 3C). Since then, many reports of swelling-induced degrafting of hydrophilic polymer brushes have been published and can be found in recent reviews.^86−,43^ Degrafting of hydrophilic brushes not only occurs upon incubation in liquid water but has also been observed upon exposure to humid air.^44^ The rate of degrafting was increased with more hydrophilic polymers and higher relative humidity. More recently, degrafting was also reported for hydrophobic poly(*tert*-butyl methacrylate) (PtBMA) brushes in organic solvents.^45^ In good, dry solvents, degrafting was not observed even when they caused swelling in the brush, but it was found that water was necessary to initiate degrafting. This is the first experimental work where the initial rate of degrafting could be correlated to the degree of swelling.

Swelling of polymer brushes has also been demonstrated to be sufficient to activate mechanophores located between the brush and substrate. Lyu et al. demonstrated the activation of a furan-maleimide Diels–Alder (DA) adduct tethering a brush to a quartz crystal microbalance (QCM) chip by salt-induced brush swelling (Figure 3D).^46^ The degree of swelling of the mechanophore-bearing polymer was enhanced with increasing salt concentration. Only at a certain degree of swelling was brush cleavage observed. It should be noted that the activation energy required for the retro-cycloaddition reaction to take place is usually rather high, indicated by the use of either ultrasonication or high temperatures.

#### Surface-Anchored Thin Polymer Films

Confinement of a cross-linked polymer thin film onto a substrate alters its swelling behavior. Such films show a smaller dependence of equilibrium swelling on the cross-linking density compared to bulk polymer gels.^35^ Swelling of a laterally confined polymer film generates anisotropic osmotic stress parallel to the film thickness, leading to the generation of a net compressive force in the film. This situation may cause surface instabilities resulting in the development of surface patterns like buckling, wrinkling, folding, or even delamination in the case that swelling-induced tension exceeds the adhesion strength of the film. This section highlights the swelling behavior of polymer films that have been confined to solid surfaces via covalent bonds.

**Figure 4:**
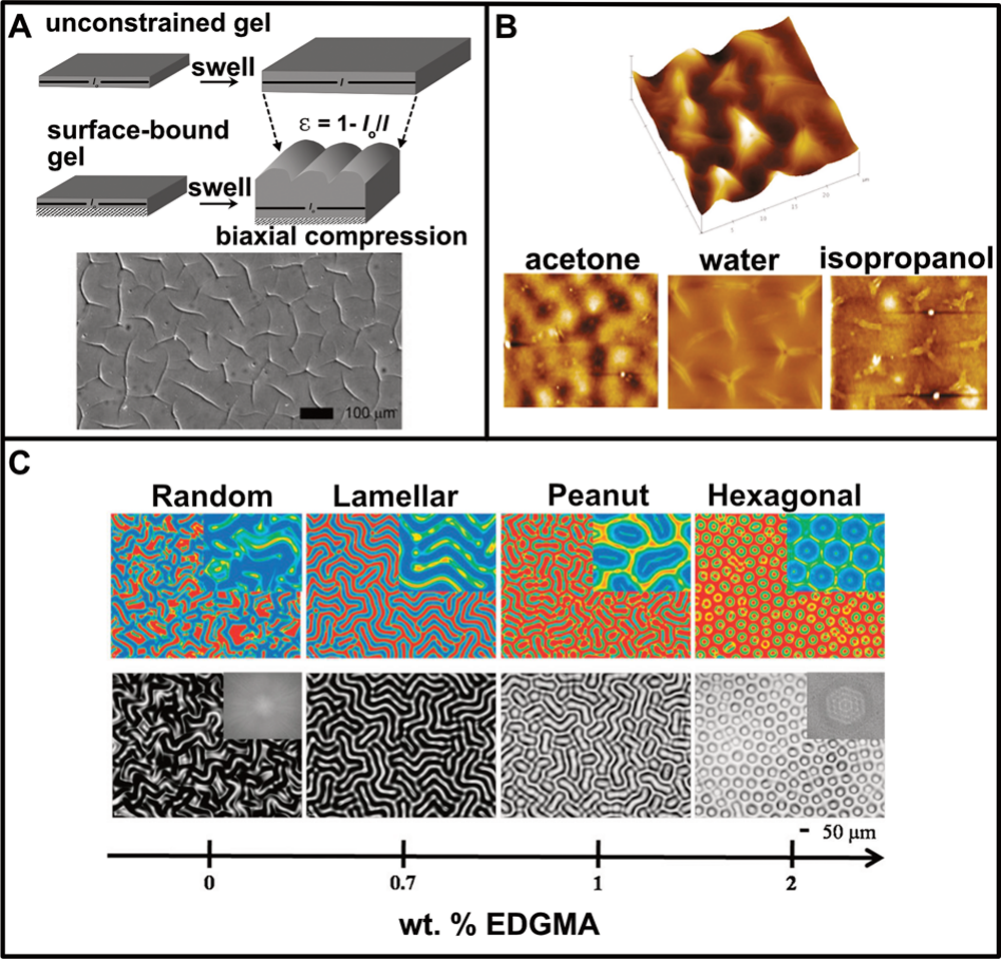
(A) (top) Schematics showing swelling-induced effects in an unconstrained surface-bound gel. Wrinkling is observed in the surface-bound gel resulting from the buildup of compressive stress. *l*_0_ and *l* are the initial and final dimension of the gels, and ε is the compressive strain. (bottom) Optical micrograph showing the patterns of creasing in poly(acrylamide-*co*-acrylate) gels (dry thickness = 60 μm) covalently bound to a coverslip when swollen in PBS. (Reprinted with permission from ref (51). Copyright 2008 Royal Society of Chemistry.) (B) AFM micrographs of poly(NIPAAm-*co*-MaBP) coatings. (top) 3D view of the cusps formed in the film after exposure to water (dry thickness = 800 nm). (bottom) Surface instabilities in the films (dry thickness = 300 nm) upon swelling in acetone, water, and isopropanol for 30 s. (Reprinted with permission from ref (52). Copyright 2010 American Chemical Society.) (C) RGB and grayscale optical micrographs of patterns formed in PHEMA films upon exposure to water. The patterns are a function of the gradient cross-linking density along the film thickness. (Reprinted with permission from ref (54). Copyright 2009 John Wiley and Sons.)

Southern and Thomas studied the effects of swelling on a vulcanized rubber film (∼1 mm) attached to a steel surface and constrained laterally by rigid plates.^47^ Swelling in a good solvent caused the development of creases, increasing with decreasing cross-linking density and improving solvent quality. The authors interpreted this observation as a surface instability caused by swelling-induced compressive strain generated in the polymer, which exceeds the material-specific critical stress. Tanaka et al. further investigated the formation and coarsening dynamics of the instability patterns in polymer films covalently attached to the underlying substrate, both theoretically^48,49^ and experimentally.^50^ The osmotic pressure developed in the film increases with time and leads to the formation of cusps, which eventually form folded structures. The shape of the folds was found to be dependent on the initial thickness and adhesion strength of the film. As some folds grow deeper and touch the underlying surface, a discontinuity in the gel expansion is generated, leading to a rupture of the gel from the surface due to the buildup of stress. Hayward and co-workers also observed the development of instability patterns (creasing) in PBS-swollen poly(acrylamide-*co*-acrylate) gels chemically bound to a coverslip (Figure 4A).^51^ They estimated that the onset of patterns occurs at a critical compressive strain of ∼0.33 or a swelling ratio of ∼2.

Similarly, cross-linked poly(*N*-isopropylacrylamide) (pNIPAM) films attached to silicon surfaces developed cusps or folds when exposed to a solvent.^52^ The morphology of surface patterns was dependent on the solvent quality, with exposure to isopropanol (a good solvent with structural similarity to the monomer) and water showing the development of characteristic folds with cusp patterns, while acetone (a relatively poor solvent) showed blisterlike patterns instead (Figure 4B). The morphology of these patterns could also be tuned by controlling cross-linking density both perpendicular^53,54^ and lateral^55^ to the substrate. These surface patterns observed in poly(hydroxyethyl)methacrylate (PHEMA) films ranged from random to lamellar to peanut-shaped to hexagonal upon varying the cross-linker (ethylene glycol dimethacrylate, EDGMA) and/or polymer concentration (Figure 4C).

The effect of the adhesion strength of a gel to an underlying substrate on instabilities was also studied. Velankar et al. found that if a polymer film is loosely bound to the substrate, swelling-induced stress can generate folds with an aspect ratio higher than the film dimensions themselves.^56^ This was caused by buckling delamination (as the compressive stress can overcome the adhesion stress) at random sites with film material eventually sliding in the fold direction to feed the growth of this irreversible fold formation. Delamination was also observed in poly(d,l-lactide) (PLA) films on silicon upon rapid swelling, causing blisterlike patterns which grew over time.^57^ Blisters are points of failure at the interface of PLA and the silicon substrate and were controlled by the film–substrate adhesion strength.

These studies highlight the effects of the stress generated in swollen polymer gels due to lateral confinement to a rigid surface. Other examples like polymer films on elastic substrates like PDMS or elastic films with a rigid top layer composed of metals or polymers also show swelling-induced effects due to the differential swelling of the bilayer. These systems are complex, as the underlying substrate also undergoes swelling, and are not covered in this Invited Feature Article. Interested readers are referred to other excellent reviews to learn more.^58−60^

### Three-Dimensional Polymer Materials

While there exist a handful of approaches to describe tension amplification of bottlebrushes in solution (1D) and tethered polymer brushes on surfaces (2D), to our knowledge, no model has been published to estimate the forces resulting from swelling of 3D polymer networks. The more complex architecture, and often observed divergence between perfect model networks and real-world defect-containing networks, renders a force estimation more challenging and is beyond the scope of this work. Swelling of a cross-linked polymer network causes the polymer chains to stretch. Depending on the cross-link density, the network architecture, and the polymer–solvent interaction, the swelling equilibrium is reached at different amounts of solvent uptake. Swelling of polymer networks, hydrogels, and organogels results in a mechanical force acting on the stretched polymer chains and cross-linking points.

**Figure 5:**
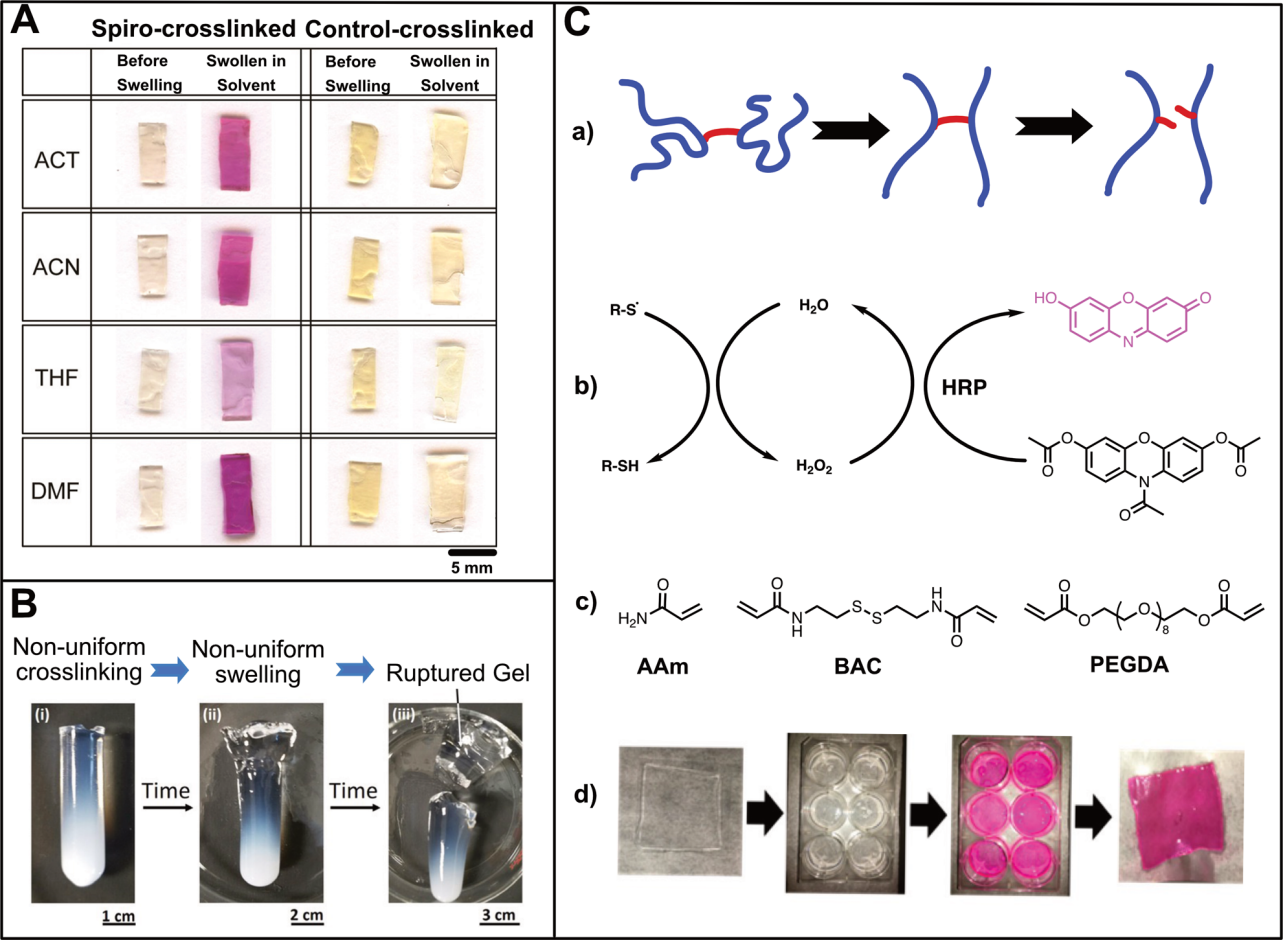
(A) PMMA samples cross-linked through spiropyran (1 mol % cross-linker) before and after swelling in acetone (ACT), acetonitrile (ACN), tetrahydrofuran (THF), and dimethylformamide (DMF); the purple color indicates the conversion of spiropyran to the colored merocyanine form. Control spiropyran cross-linked PMMA samples swell similarly to the spiro-cross-linked samples but display no color change unless irradiated by UV light (shown for acetone-swollen control sample only). Only the bottom part of the sample was irradiated with UV. (Reprinted with permission from ref (23). Copyright 2014 American Chemical Society.) (B) Nonuniform swelling of regions of different cross-link densities results in complete rupture at the interface. (Reprinted with permission from ref (65). Copyright 2016 American Chemical Society.) (C) (a) Scheme of bond breakage in hydrogels under mechanical tension. As gels swell, their chains extend, generating mechanical tension in the short cross-linkers. (b) The mechanochemical breakage of thiols results in the abstraction of a hydrogen radical from ambient water, producing hydrogen peroxide. The hydrogen peroxide converts colorless Amplex Ultrared (AUR) to pink resorufin. (c) Hydrogel components utilized in this study: polyacrylamide (AAm), *N*,*N*′-bis(acryloyl)cystamine (BAC), and polyethylene(glycol) diacrylate (PEGDA, *M*_w_ = 400). (d) Photographs of the process. The polyacrylamide hydrogel is prepared as a thin square and then placed in a buffer containing AUR and HRP. The resorufin equilibrates with the external fluid, which is sampled and its absorbance measured. (Reprinted with permission from ref (68). Copyright 2018 American Chemical Society.)

Swelling-induced forces in polymer networks have been visualized using mechanophores that are incorporated within the network. Spiropyran embedded in a cross-linked poly(methyl methacrylate) (PMMA) matrix undergoes a ring-opening isomerization reaction to a colored merocyanine form, by swelling in different solvents including acetone (ACT), acetonitrile (ACN), tetrahydrofuran (THF), and dimethylformamide (DMF) (Figure 5A).^23^ A direct correlation between the degree of cross-linking, hence the degree of swelling, and the fluorescence intensity was observed. Control experiments in which the spiropyran was differently embedded into the polymer chain such that the force did not directly act on the weak bond showed no activation, proving the force-induced nature of the ring-opening.

Kim et al. studied poly(dimethylsiloxane) (PDMS) networks containing spiropyran mechanophores.^61^ The prepared films were pre-swollen in xylene and tested under both uniaxial tension (tensile testing) as well as compression and bending with simultaneous measurement of the fluorescence intensity. It was found that swelling induces a pre-strain in the network, which decreased the fluorescence-activation onset strain. The mechanosensitivity of the samples increased linearly with swelling time. However, directly after swelling, a decrease in initial fluorescence is observed as the spiropyran form is preferred over the merocyanine form in the nonpolar xylene. The pre-strain, induced only by swelling, was therefore not sufficient to activate the ring-opening reaction.^61^ It is interesting that in this example an external mechanical force was necessary to activate the pre-strained material, while for Lee et al, swelling was sufficient for mechanophore activation. One explanation may be the position of the mechanophore in the polymer network. While the spiropyran molecules were randomly distributed in the PDMS network of Kim et al., Lee et al. used spiropyran-containing cross-links, which can experience significantly higher stress upon swelling. The localization of the mechanophore within the polymer network is, therefore, an important consideration. On the other hand, Kim et al. only tried swelling in xylene, and the behavior of this system in more polar solvents is so far unknown. Spiropyran activation of microgels upon swelling was published by Li et al. In this case, swelling was not induced by solvent uptake but by CO_2_ aeration.^62^ Microgels were prepared from 2-(dimethylamino)ethyl-methacrylate (DMAEMA) and a low-molecular-weight spiropyran-containing cross-linker. The change from spiropyran to merocyanine was monitored with confocal fluorescence microscopy and was reversible upon washing with nitrogen. The reason for the CO_2_-swelling is probably the higher protonation degree of the PDMAEMA amino groups in the presence of CO_2_, which results in increased water uptake due to increased osmotic pressure.

Another example of mechanophore activation within an organogel induced by solvent swelling was published by Clough et al.^63^ In this study, solvent swelling of a PMMA network containing bis(adamantyl)-1,2-dioxetane cross-links was studied. The 1,2-dioxetane mechanophore splits into two ketones in the excited state upon application of a mechanical force. These excited ketones exhibit chemiluminescence. Clough et al. reported the swelling of these mechanophore-bearing PMMA networks in different solvents, including chloroform. Upon swelling, a photodiode captured the chemiluminescence, and therefore, the mechanical cleavage of the mechanophore. Control experiments, in which bis(adamantyl)-1,2-dioxetane was dispersed into the polymer network, but not covalently connected, did not show any chemiluminescence. Further, they observed sample cracking at high swelling degrees, indicating that the swelling-induced force is sufficient to alter the micro- as well as the macroscopic architecture of the network.

Recently, Watabe and Otsuka reported a swelling-induced mechanochromism phenomena in a multinetwork hydrogel.^64^ The first network of the hydrogel was synthesized by photopolymerization of ethyl acrylate with mechanochromic difluorenylsuccinonitrile (DFSN) dimethacrylate cross-linker. DFSN responds to changes in the structure of the hydrogel network by undergoing a reversible C–C bond cleavage to yield pink cyanofluorene radicals. This network was reswollen in the monomer and a non-mechanochromic cross-linker solution for subsequent polymerization steps to yield a multinetwork hydrogel. The authors hypothesized that repeated expansions and photocuring steps of the first network led to lowering of the threshold force required to activate the DFSN mechanophore in its network, such that it could be activated by forces generated during swelling of the hydrogel. The study found that a triple network hydrogel showed higher mechanochromism (more intense color) than a double network hydrogel when swollen in various solvents. Furthermore, the authors reported that the swelling-induced network stretching was isotropic, and the threshold swelling degree required for DFSN activation was 1.15–1.20 for a triple network hydrogel.

Macroscopic alterations, leading to complete rupturing of polymer networks, were observed by de Silva et al.^65^ They prepared self-rupturing hydrogels from poly(acrylic acid) (PAA) via covalent cross-linking with methylenebis(acrylamide) (MBA). The self-rupturing effect was imposed by nonuniform swelling of regions with different cross-link densities (Figure 5B). The interfacial stresses between these regions became sufficiently high to finally rupture the gel completely from the region with high swelling ratio (low cross-linking density). Although no mechanophore was introduced in these materials for visualization, the authors concluded that the rupture was caused by swelling-generated forces that were high enough to break covalent bonds supporting the hydrogel network.

Swelling-induced mechanochemical effects have also been observed in water-containing cross-linked polymer networks, i.e., hydrogels. Shah et al., as an example, prepared hydrogels by photopolymerizing poly(lactic acid)-*b*-poly(ethylene glycol)-*b*-poly(lactic acid) diacrylate (PLA-*b*-PEG-*b*-PLA) triblock macromers (also known as macromonomers) (*M*_n_ around 4600 Da) and studied the degradation behavior of the hydrogel upon hydrolysis of PLA ester bonds.^66^ They revealed a relationship between hydrogel degradation and water concentration, macromer concentration, pH value, and ionic strength. It was found that the pseudo-first-order rate constants for degradation of soluble macromers increase with water concentration; in other words, the degradation rate constants displayed a positive correlation with the swelling ratio of hydrogels.

Wang et al. developed a rhodamine (Rh) mechanophore cross-linked poly(NaAMPS-*co*-DMAAm)/PAAm double network hydrogel.^67^ Similar to spiropyran, Rh undergoes an isomerization reaction from a twisted spirolactam form (nonfluorescent) to a planar zwitterionic form (fluorescent), with corresponding emission bands at 440 and 558 nm. When covalently integrated into a hydrogel network, the Rh mechanophore was effectively activated upon application of an external force (stretching). The authors, however, also observed a residual activation of Rh in the swollen unstretched hydrogel network, which they attributed to the swelling-induced ring-opening of a few Rh molecules.

Another interesting work published by Goodwin et al. aimed at investigating the swelling-induced mechanochemistry of hydrogels (Figure 5C).^68^ Polyacrylamide hydrogels were synthesized with acrylamide (AAm) and *N*,*N*′-bis(acryloyl)cystamine (BAC). Since BAC contains a disulfide bond, the mechanochemical breakage of thiols results in the abstraction of a hydrogen radical from water, producing hydrogen peroxide (H_2_O_2_). To visualize this process, horseradish peroxidase (HRP) and Amplex Ultrared (AUR) were used. With the catalysis of HRP, H_2_O_2_ can convert colorless AUR to pink resorufin. Besides BAC, they also tested hydrogels cross-linked with poly(ethylene glycol) diacrylate (PEGDA), which is considered a stronger cross-linker, whose dissociation energy is one-third higher than a disulfide bond. It is found that hydrogels with stronger cross-linkers showed a decreased H_2_O_2_ production compared to hydrogels with weaker cross-linkers. When the gels were bound to the surface, the constrained 1D swelling generated stress enough to also cleave C–C and C–O bonds in the gels cross-linked with PEGDA resulting in increased H_2_O_2_ generation as compared to its unbound state. However, constrained swelling of gels with BAC cross-links did not show a significant increase in the H_2_O_2_ production compared to the unbound gel. The gel swelling alone proved to be sufficient to generate H_2_O_2_ in weaker gels.

## Visualization and Quantitative Characterization of Swelling-Induced Mechanochemical Activation of Polymeric Materials

**Figure 6:**
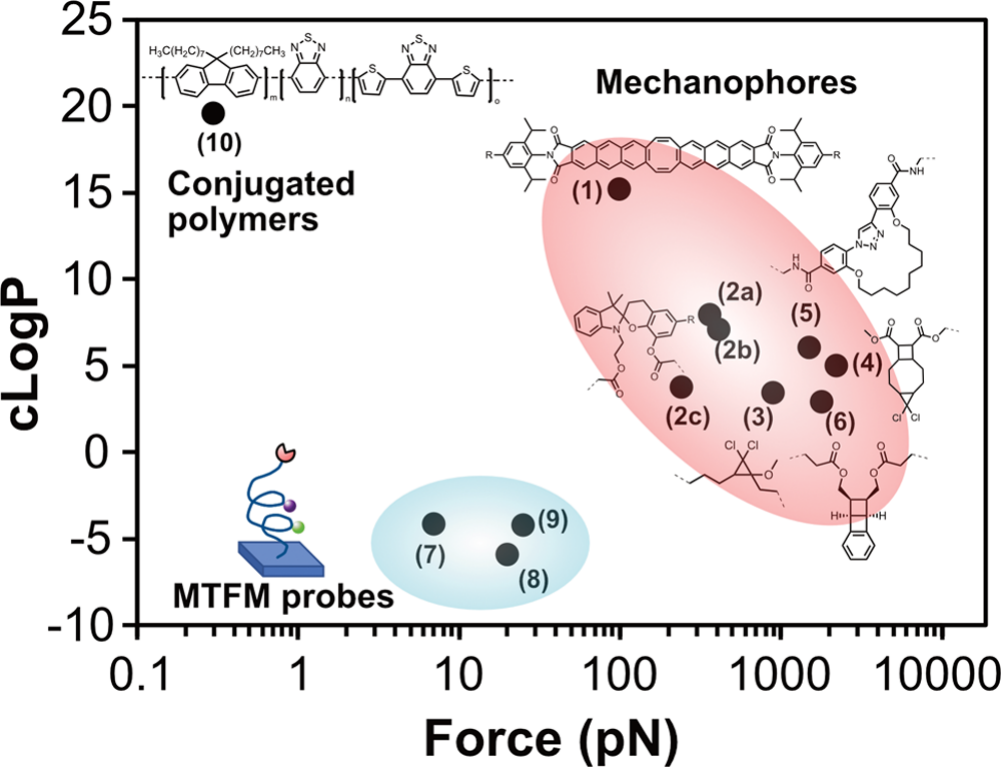
cLogP vs force plot for several selected force probes and mechanophores. Chemical structures of mechanophores (red circle): (1) Flapping molecule or FLAP probe.^70^ (2) Spiropyran derivative with R as −Br (2a), −H (2b), and −NO_2_ (2c).^71^ (3) 2-Methoxy substituted *gem*-dicyclopropane (MeO-*g*DCC).^72^ (4) 5,5-Dichlorotricyclo(7.2.0.0)undecane (DCTCU).^73^ (5) 1,2,3-Triazole-based macrocycle.^74^ (6) Benzo-ladderane mechanophore.^75^ Cartoon depiction of MTFM probes (blue circle) based on (7) FRET with (GPGGA)_8_ peptide sequence as linker,^76^ (8) FRET with PEG_24_ as linker,^77^ and (9) NSET with PEG_80_ as linker.^78^ (10) Conjugated polymer system.^85^

The macroscopic deformations in swollen polymeric materials emanate from molecular-scale swelling-induced stresses. Visualizing and quantitatively assessing these forces is essential to understand the mechanochemistry of polymers that are exposed to good solvents. To this end, mechanophores are needed that respond to a wide range of forces, ideally with a change in color, which allows visible or spectrophotometric analysis.^69^ With the mechanophores that are currently available, this represents a significant challenge. To illustrate this, Figure 6 presents an overview of various mechanophores and force probes, plotting both the force/force ranges that these moieties respond to as well as their partition coefficients (reported as cLogP, and estimated by ChemDraw) as an indicator of their polarity (higher cLogP values indicate more nonpolar compounds). As illustrated in Figure 6, there are several mechanophores that are well-characterized and that respond to forces in the range of 100–1000 pN. The force response of these mechanophores has been validated using single-molecule force spectroscopy (SFMS), which allows these to be incorporated as force probes in polymer-based systems. Some of these mechanophores respond to forces by undergoing a bond-breakage or rearrangement accompanied by a change in color, which may enable visible or spectrophotometric observation of swelling-induced mechanochemical effects. The limitations of the force probes that have been used so far is that these (i) respond only to a limited range of forces and (ii) utilize non-water-soluble probes, potentially impacting their application to the study of hydrogels. As a consequence, there is a need for molecular force probes that respond to a broader range of forces, allow for quantitative force mapping, and are applicable for the use in aqueous media.^6^ The remainder of this section will highlight some of these techniques/probes that can be used as potential tools to study water-swollen polymer systems.

**Figure 7:**
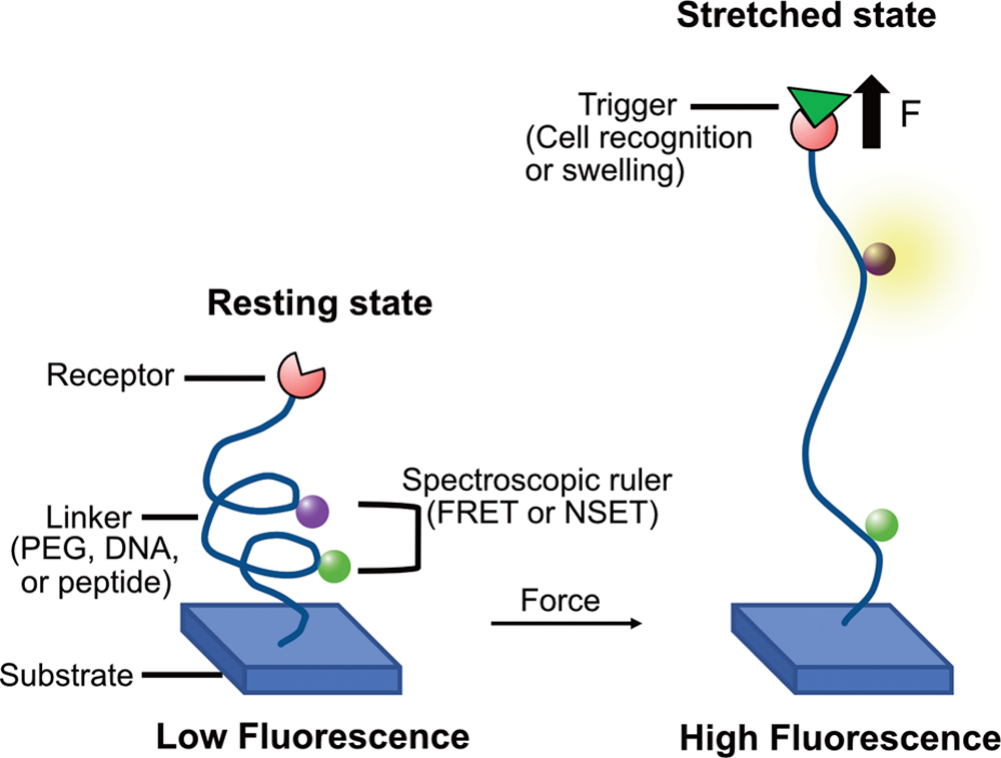
Depiction of molecular tension fluorescence microscopy (MTFM) with a probe in resting and stretched states. Redrawn with permission from ref (79). Copyright 2017 American Chemical Society.

The biophysical community has been developing a number of experimental techniques such as, for example, traction force microscopy (TFM) and single-molecule force spectroscopy (SMFS) that are well-suited to interrogate weak forces in aqueous media.^79,80^ While TFM measures forces that are orders of magnitude higher than forces found in biological systems and most soft materials, SMFS-based techniques can sense forces down to several pNs but suffer from low throughput. Another technique, called molecular tension fluorescence microscopy (MTFM), has been pioneered by the Salaita lab and successfully used to map and quantify cellular traction forces.^79,81^ MTFM employs tension probes consisting of a flexible linker that connects two chromophores acting as a spectroscopic ruler (Figure 7). The choice of a flexible linker comprises polymers like poly(ethylene glycol) (PEG), DNA hairpins, or α-helical and β-sheet peptides that show a force–displacement behavior. Spectroscopic rulers can range from Förster resonance energy transfer (FRET) requiring chromophore pairs to nanometal surface energy transfer (NSET) requiring chromophore and gold nanoparticles. These probes are immobilized on a substrate using either affinity binding (e.g., streptavidin–biotin coupling), chemisorption (self-assembly of thiolated probes on gold surfaces), or covalent binding (click chemistry or halo-tag ligation).

The fluorescence intensity of MTFM probes varies with the distance between the chromophore and can be computed using fluorescence microscopy. In the resting state, the fluorescence is maximally quenched by the energy transfer between the chromophores. However, if a traction force stretches the polymer chain and the chromophores separate, the fluorescence intensity increases.^79−82^ Quantification of forces is possible after acquisition of a force–fluorescence calibration curve, for which the force–elongation curve of the flexible linker also needs to be known (which is accessible with quantum mechanical calculations or SMFS).^81,83^ Immobilized PEG-based probes with FRET dyes encompass the dynamic force range of 20 pN while the PEG MTFM probes based on the NSET energy transfer mechanism show up to a 10-fold increase in fluorescence intensity and achieve a force range of 27 pN.^77,78^ Similarly, DNA hairpins used as flexible linkers can achieve a 20- to 100-fold increase in the fluorescence intensity with FRET and the NSET-based energy transfer mechanism, respectively.

Currently, MTFM probes have been mainly utilized for measuring biomechanical forces, but these could potentially be adapted for measuring weak forces in polymeric materials. Besford et al. reported a very first example of using MTFM to study the conformational changes of poly(*N*-isopropylacrylamide) (PNIPAM) polymer brushes containing FRET-pairs upon swelling in water/alcohol mixtures.^84^ However, no quantitative information about the swelling-induced range of forces was given.

Sprakel and co-workers recently reported another molecular force probe, which allows the detection of forces at a sub-piconewton level.^85^ The probe, which is based on a doped semiconducting polymer backbone, consists of poly(fluorene-*alt*-benzothiadiazole) (F8BT) donor monomers and a few dithenyl benzothiadiazole (DTBT) acceptor molecules as dopants. The probe was embedded in a polystyrene (PS) film subjected to mechanical deformation, and SMFS was used to estimate corresponding changes in fluorescence of the probe. The different degrees of energy transfer between the acceptor and donor molecule were indicative of the corresponding degrees of stretching of the probe chains embedded in the PS matrix, and the probe allowed for quantitative mapping of the weak forces as low as ∼300 femtonewtons. The probe also responded well to the presence of good or poor solvent as the collapsed chains showed pure acceptor emission due to enhanced energy transfer, while increased emission from the donor was observed as the polymer chains swelled in a good solvent.

## Conclusions

Weak forces play an important role in biology, but have received less attention with regards to their impact on synthetic polymer systems. This Invited Feature Article attempted to depict the generation of weak mechanical forces in polymeric materials at multiple dimensions upon solvent swelling. Quantification of low-range forces is still a challenge due to a lack of relevant techniques. The few available techniques like SMFS, TFM, etc., are complicated and require advanced expertise. While biologists have developed and extensively used tools like MTFM to measure weak forces exerted by cells, polymer scientists have just started to quantitatively measure weak mechanical forces that act on polymer materials and to assess the macroscopic impact of these forces. MTFM is a promising technique that is also of interest for force quantification in polymeric materials. For example, MTFM could be applied to polymer hydrogels, which find applications as a matrix in tissue engineering and are sensitive to the mechanobiology of cells. Furthermore, there is a demand for the development of water-soluble mechanophores. Currently, FRET or NSET-based probes used in MTFM are the only known examples of water-soluble mechanophores; however, none of these have been employed to quantify forces in hydrophilic polymer systems. The mechanochemical aspects of swelling-induced phenomena in polymeric materials are crucial for understanding molecular-level solvent–polymer interactions and being able to design a new generation of responsive materials. Further efforts are therefore required to expand the range of detectable forces by designing improved force sensors, with a broad level of applicability in synthetic polymeric materials.
